# Tactics for mechanized reasoning: a commentary on Milner (1984) ‘The use of machines to assist in rigorous proof’

**DOI:** 10.1098/rsta.2014.0234

**Published:** 2015-04-13

**Authors:** M. J. C. Gordon

**Affiliations:** University of Cambridge Computer Laboratory, William Gates Building, 15 JJ Thomson Avenue, Cambridge CB3 0FD, UK

**Keywords:** theorem proving, formal verification, functional programing, types

## Abstract

Robin Milner's paper, ‘The use of machines to assist in rigorous proof’, introduces methods for automating mathematical reasoning that are a milestone in the development of computer-assisted theorem proving. His ideas, particularly his theory of tactics, revolutionized the architecture of proof assistants. His methodology for automating rigorous proof soundly, particularly his theory of type polymorphism in programing, led to major contributions to the theory and design of programing languages. His citation for the 1991 ACM A.M. Turing award, the most prestigious award in computer science, credits him with, among other achievements, ‘probably the first theoretically based yet practical tool for machine assisted proof construction’. This commentary was written to celebrate the 350th anniversary of the journal *Philosophical Transactions of the Royal Society*.

## 1. The paper's history and its importance at the time

Robin Milner first became interested in using machines to perform proofs while working as a research assistant for David Cooper at Swansea University. In an interview with Martin Berger in 2003 [1], Milner says ‘I wrote an automatic theorem prover in Swansea for myself and became shattered with the difficulty of doing anything interesting in that direction’. Influenced by ideas of Floyd [2] and Hoare [3], Milner tried to use his theorem prover to verify the correctness of programs by generating logic formulae with the property that if the formulae are true then the programs from which they were generated are correct. Quoting again from Berger's interview: ‘I generated verification conditions from programs. But then it took ages to prove them. My theorem-prover couldn't do it’. At that time, Robinson's resolution method for automatically proving first-order logic formulae [4] was very influential. Berger quotes Milner as saying ‘I greatly admired Robinson's resolution principle, a wonderful breakthrough, but, in fact, the amount of stuff you can prove with fully automatic theorem proving is still very small. So, I was always more interested in amplifying human intelligence than I am in artificial intelligence’. In 1970, Milner had an opportunity to explore this interest at the Stanford Artificial Intelligence Laboratory, where he took a job as a research associate working for John McCarthy.

### (a) Stanford logic of computable functions

Before moving to Stanford, Milner had visited Oxford to hear Dana Scott speak on his theory of domains. This theory was creating enormous excitement as, in combination with earlier ideas of Strachey [5], it provided a rigorous foundation for a new approach to the mathematical semantics of programing languages [6]. Scott had also invented a logic of domains for reasoning about recursive functions [7] and Milner ‘realised that I could write down the syntax of a programing language in this logic and I could write the semantics in the logic’ [1]. At Stanford, helped by Richard Weyhrauch and Malcolm Newey, Milner implemented a system that partly automated reasoning in Scott's logic, which he called LCF as an abbreviation for logic of computable functions. This subsequently came to be called Stanford LCF [8] to distinguish it from later LCF systems. Because he had come to believe that fully automatic proof was not practical, Stanford LCF was an interactive user-guided system. In contrast with the Floyd–Hoare verification condition approach experimented with at Swansea, programs could be reasoned about directly via their semantics encoded in Scott's logic. Newey demonstrated this in his PhD on the verification, using Stanford LCF, of an interpreter for pure LISP.

Stanford LCF is based on goal-directed reasoning, which was influential in artificial intelligence [9] at the time. The user states a formula as the goal to be proved and then interactively solves it by issuing commands to the system to decompose goals into subgoals. This is called *backward proof* to contrast it with *forward proof* in which rules of inference are applied to axioms until a theorem solving the desired goal is reached. Stanford LCF has a fixed set of commands for backwards proof; some, such as induction, correspond to inference rules of Scott's logic (the induction command splits a goal into two subgoals: the basis and the induction step). Other commands, such as invoking the simplifier, are complex and partly heuristic.

### (b) Edinburgh logic of computable functions

Stanford LCF was a landmark in the automation of proof. It led to and inspired the extraordinarily influential ideas that Milner expounded in the paper, which describes a methodology that addresses two issues with the system: (i) the lack of any way for users to add new proof commands, and (ii) large proofs exhausting available memory. The theory of tactics presented in the paper is Milner's solution to these issues. It was implemented in a successor system called Edinburgh LCF (or just LCF) [10] that Milner built after he moved to a permanent academic position at the University of Edinburgh in 1973.

The construction of Edinburgh LCF was funded by the UK Science Research Council. This project initially employed Malcolm Newey and Lockwood Morris, whom Milner had met at Stanford, followed by Mike Gordon and Chris Wadsworth. Two of Milner's students obtained PhDs using LCF: Avra Cohn on the verification of programing language compilers and Brian Monahan on the mechanization of datatypes.

The LCF approach described in Milner's paper had—and continues to have—a major impact on the architecture of proof assistants. In 2014, 30 years after the publication of the paper, Avigad and Harrison write in a survey of the field that many of these systems ‘are based on an architecture developed by Robin Milner with his 1972 LCF proof checker, which implemented Dana Scott's Logic of Computable Functions’ [11].

Milner's paper starts by illustrating his general theory of goal-seeking with an everyday non-mathematical example. He then shows how this theory is used for theorem proving in LCF. A proof of a theorem about parsing, done with his student Avra Cohn, illustrates the ideas in more depth. Within just ten pages, the core ideas underlying many modern proof assistants appear. These include (i) using a functional programing language [12] as a proof assistant metalanguage, (ii) representing terms, formulae and theorems of a logic—the object language—as metalanguage data types, (iii) using programing language types to ensure that the only way to create theorems is by proof, (iv) the concept of tactics for representing subgoaling strategies as metalanguage functions, and (v) the use of higher-order functions for combining tactics. These ideas were quickly recognized as major methodological advances and almost immediately inspired three projects to implement proof assistants: by Petersson at Chalmers University in Sweden [13], by Constable at Cornell in the USA [14] and by Huet and Coquand at INRIA in France [15]. These projects were for different object languages, all versions of constructive-type theory, and the systems to which the latter two led, Nuprl and Coq, are still used today.

The language ML, originally designed as a proof metalanguage for LCF, had a major impact on the field of programing languages. The citation for the 1991 ACM Turing Award [16] states that, in addition to Milner's contribution to machine assisted proof, the award is also for: ‘the first language to include polymorphic type inference together with a type-safe exception-handling mechanism’. It is a testament to Milner's design skill that his metalanguage for interactive proof has had an impact far beyond theorem proving. His theory of types [17], first implemented in ML, has been admired, adopted and extended, first in functional programing, and then in other kinds of language (e.g. in the type systems of the object-oriented languages Java and C#).

## 2. Milner's theory of tactics

The core idea of Milner's theorem proving method [18]—the LCF approach—is to use programing language functions, which he called *tactics*, simultaneously to reduce a goal to subgoals and, crucially, to generate a procedure, called a *validation*, to convert solutions of the subgoals into a solution of the goal. The tactic concept is intended to be part of a theory that is more general than mathematical theorem proving. To reflect this generality, Milner introduces the term *event* to represent things that achieve (i.e. solve) goals. This terminology may have come from the everyday example in the first section of the paper in which goal achievements were occurrences of people at a particular time and place. In addition to goals G and events E, Milner's theory assumes that a relation of achievement between them is specified: the notation ‘E achieves G’ means that the event E achieves the goal G. Milner defines^1^ the set tactic of tactics by the equation

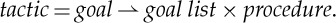

The set tactic is thus equal to the set of partial functions from the set goal to the product of the sets goal list and procedure. If T∈tactic and G∈goal, then T(G) may or may not be defined. If it is defined, then T(G)=([G_1_;⋯ ;G_*n*_],P), where [G_1_;⋯ ;G_*n*_] denotes the list of subgoals G_1_,…,G_*n*_, and P is a procedure that validates the splitting of the goal into subgoals by providing a way to get an event solving the goal G from events solving the subgoals. The validation procedure P is a member of the set procedure, where

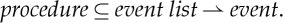

Thus, P∈procedure and so is a partial function from event list to *event*. A tactic T is called *valid* by Milner if whenever T(G)=([G_1_;⋯ ;G_*n*_],P), and if E_*i*_ achieves G_*i*_ for all *i* such that 1≤*i*≤*n*, then *P*([E_1_;⋯ ;E_*n*_]) is defined and is an event that achieves G. If the list of subgoals is empty, i.e. T(G)=([ ],P), then T is said to *solve* the goal G and P([ ]) should then evaluate to an event that achieves G.

In the early days of Edinburgh LCF, users interactively applied tactics to goals and then to subgoals until all subgoals were solved. The validation procedures were then manually applied in the reverse order to compute events that achieved the subgoals and, eventually, the main goal. For example, a user might manually solve a goal G and all subgoals generated from it by applying tactics T_1_, T_2_, T_3_ and T_4_ in turn, as shown in the left column of the box given here.

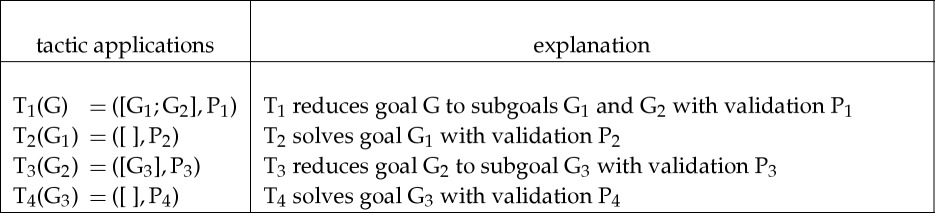

After applying tactics T_1_, T_2_, T_3_ and T_4_ as above, the user then applies the generated validation procedures P_2_, P_4_, P_3_ and P_1_, as shown below, to get the event E_4_ that solves G.

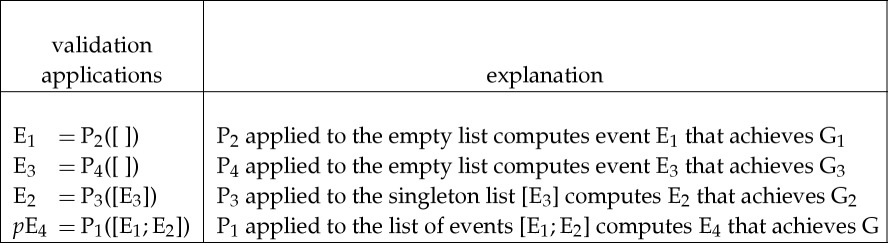

The actual production of subgoals, and the subsequent computation of events to achieve them, was done by interacting with a read–eval–print loop in which the user inputs an ML expression and the system then reads, evaluates and prints out its value. The computation of the event to achieve G could either be done in four steps to separately and successively compute E_1_, E_2_, E_3_ and E_4_, as above, or by a single evaluation of the expression P_1_([P_2_([ ]);P_3_([P_4_([ ])])]) to compute E_4_ in a single step.

### (a) Tacticals

Unlike users of Stanford LCF, users of Edinburgh LCF can write programs to perform sequences of actions automatically. The metalanguage ML was specifically designed for writing such programs. For example, in ML, one could define a single tactic T that combines T_1_, T_2_, T_3_ and T_4_:

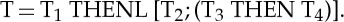

THENL and THEN are *tacticals*, Milner's name for operators that combine tactics. Tactical THENL applies its left argument, tactic T_1_, to a goal and then applies each tactic in its right argument, a list containing two tactics T_2_ and (T_3_ THEN T_4_), to the two goals returned by T_1_. Tactical THEN applies its left argument T_3_ to the second goal produced by T_1_ and then applies T_4_ to all the goals produced by T_3_. Both THENL and THEN concatenate the subgoals they produce into a flattened list of subgoals and combine the validation functions appropriately.

If a tactic is applied to a goal for which it is not suited, e.g. an induction tactic is applied to a goal that cannot be decomposed into basis and step subgoals, then the inapplicability is signalled by the application of the tactic to the goal *failing*. The LCF metalanguage ML supports such failure via an exception-handling mechanism. The infixed tactical ORELSE, described in §2 of the paper, uses this exception mechanism to try a tactic and, if it fails, to try an alternative one. The tactical REPEAT repeatedly applies a tactic until it fails.

### (b) Saving space by not storing proofs

Another motivation for the invention of tactics was to reduce the space taken up by proofs. A sequence of interactions with Stanford LCF creates a proof tree stored in the computer's memory. The effect of each command is to expand this tree by adding the subgoals generated.

In the early 1970s, computers had much less memory than now, and thus large proofs might exhaust what was available. The Digital Equipment Corporation KA10 PDP-10 mainframe, which was used at the Stanford AI Laboratory where Stanford LCF was developed, had 196 608 36-bit words of memory [19], i.e. around 1 MB, less than 0.1% of the RAM of a typical 2014 smartphone.

With Edinburgh LCF, Milner wanted to preserve the soundness guarantee that every theorem produced using the system had a proof, but he wanted to avoid having to store actual proofs. The validation procedure mechanism in his theory of tactics is his way to achieve this.

### (c) Using types to ensure logical soundness

Section 2 of Milner's paper describes the instantiation of his general theory of goal-seeking (described in §1 of the paper) to theorem proving. In this instantiation, goals are pairs (*Γ*,G), where *Γ* is a set of logical formulae—the assumptions—and G a formula that one wants to show follows from the assumptions. Events are theorems Δ⊢F where, slightly simplifying, Δ⊢F achieves (*Γ*,G) is defined to mean Δ⊆*Γ* and F=G, i.e. the conclusion of the theorem is the goal formula G and this is proved to follow from a subset Δ of the assumptions *Γ*.

Both goals and events (theorems) are represented in the LCF metalanguage ML by pairs with first component a list of formulae (the assumptions) and second component a formula (the conclusion). The difference between a goal and a theorem is that the latter has been proved from axioms using rules of inference. Milner's brilliant solution to making and enforcing this distinction between goals and theorems is to use a type discipline to keep them apart. All values in ML have a type. Goals have type (*formula* list)×*formula*, whereas theorems have an *abstract-type* *theorem* that has the same underlying representation as goals, but which the ML type-checker treats differently by ensuring that only functions representing sound inference rules can create new theorems, i.e. values of the type *theorem*. The primitive operations on the abstract type of theorems represent the axioms and rules of Scott's logic, so the effect of this type discipline is that the only way to create new theorem values is to apply compositions of inference rules to existing theorem values, which can only be axioms or previously proved theorems.

If the example in the boxes above corresponded to theorem proving, then P_2_ and P_4_ would generate instances of axioms when applied to the empty list [ ], whereas P_3_ and P_1_ would be inference rules: P_3_ having one hypothesis and P_1_ having two.

In Stanford LCF, there were no validation procedures, so the user just had to trust that the subgoaling algorithms were implemented soundly. In Edinburgh LCF, Milner wanted to allow users to program their own subgoaling algorithms by defining their own tactics and tacticals.

The validation functions produced by tactics create theorems, and Milner wanted to ensure that users could not program unsound theorem-generating functions. He writes

[…] we draw attention to a point of great pragmatic significance. Our metalanguage allows a user to write tactics – even invalid ones – with great freedom. But with this freedom he can by no means generate an event that […] is not a theorem. This follows from the fact that events are objects of type theorem, and that the only operations for generating them are the basic inference rules […] and rules derived from them. This is a fine illustration of the security provided by a type discipline; indeed, without it we could not claim to present a viable methodology.

It is the ability of LCF system users to safely program tactics without worrying about soundness that make the tactic approach so attractive, and it is one of the aspects of LCF that influenced subsequent work.

## 3. From 1980 to 2014

After the original funding for Edinburgh LCF finished, Milner procured a successor grant jointly with Mike Gordon, who had moved to the University of Cambridge Computer Laboratory in 1991. Larry Paulson was hired as the Research Assistant at Cambridge in 1982. He extended the LCF logic by adding all the standard constructs of predicate calculus (Scott's logic lacked both disjunction and existential quantification) and implemented additional infrastructure to support flexible rewriting and tactic programing. The result was Cambridge LCF [20]. Paulson performed substantial demonstrator proofs using this, notably a verification of a unification algorithm.

During the 1980s, three proof assistants for constructive logic inspired by Milner's proof mechanization ideas were being developed: Petersson's ‘Programming System for Type Theory’ [13], Nuprl [14] and Huet and Coquand's ‘Constructions’ [15]. These were all initially implemented by building on the LISP implementation of ML in Edinburgh LCF. They all used ML as a programing language for writing tactics, though Milner's original tactic concept was modified to validate goals with representations of proofs rather than theorems. A fourth system, HOL, for higher-order logic, was derived by Mike Gordon from the LISP code implementing Cambridge LCF. It used Milner's original notion of tactics, but adapted for classical higher-order logic rather than for Scott's logic [21,22].

The ad hoc method of implementing proof assistants by modifying the LCF system code inspired Paulson to create Isabelle [23], a generic tool that provides a way to create bespoke proof assistants by instantiating a logical framework, rather than by hacking the LCF implementation. Isabelle has tactics and tacticals, but they work differently from those in LCF as they operate on proof states rather than on subgoals [24].

### (a) Development of tactics

Scott's logic was very successful for formalized reasoning about program semantics, but it was less suitable for other applications being studied at the time (e.g. modelling and verifying hardware and checking general mathematical proofs) and so the LCF system was not further developed. It is tactics and the use of a typed programmable metalanguage for enforcing the logical soundness of theorem proving algorithm implementations, rather than the LCF logic, that have had a major impact on modern theorem proving.

Milner's original notion of tactics reduces goals to subgoals. The generated validation procedures go forward, mapping events to events. Validations of tactics composed via tacticals are compositions of the validations of the composed tactics. In LCF, where events are theorems, validations are inference rules. Using tacticals to compose tactics to create validations is one way to combine inference rules to create *derived rules*, but such forward inference rules can also be programed directly in ML starting from the axioms and using the primitive rules of Scott's logic.

During the 1990s, many theorem proving algorithms, including the resolution method that Milner implemented at Swansea, and several decision procedures, were programed as derived rules using combinations of LCF-style forward and backward proof. It became clear that Milner's programing methodology enabled pretty much any kind of formal reasoning algorithm to be mechanized in a guaranteed-sound way. However, programing algorithms as derived rules or tactics is usually more complicated than programing them directly, and the resulting implementations sometimes had poor performance.

In the early 1990s, the PVS proof assistant [25] emerged from SRI. PVS is based around a set of proof commands that can invoke powerful built-in algorithms for proving theorems automatically. It was not based on Milner's ideas and required less human effort to prove theorems. Another influential proof assistant was ACL2 [26], which originated from work by Boyer and Moore in the early 1970s that preceded LCF [27]. ACL2 can prove theorems automatically that require many steps of manual interactive proof in tactic-based provers. Although the core heuristics underlying the Boyer–Moore theorem prover have been implemented as a tactic in HOL [28]—just the sort of thing Milner had in mind when devising ML as a programing metalanguage for theorem proving—the resulting implementation did not compete with ACL2's generality or performance.

For at least the past 20 years, there has been an ongoing discussion about whether the performance penalty of LCF-style theorem proving is worth the logical soundness assurance that it provides. Although this debate has not been definitively resolved, descendants of Milner's LCF approach underlie many, perhaps the majority, of proof assistants today.

### (b) Progress in machine checked proofs

The proof assistants Coq, HOL^2^ and Isabelle have all built on Milner's ideas for goal-oriented proof. They originated in the 1980s and are still evolving and have growing numbers of users. Each has been used both for applications to check pure mathematics and to prove theorems that establish the correctness of hardware and software.

The best known mathematical proof to have been checked with a tactic-based proof assistant is probably the four colour theorem, which was checked with Coq [29]. An even more remarkable achievement using Coq is checking the Feit–Thomson theorem on the classification of finite groups [30]. Another impressive example uses Isabelle to prove Gödel's second incompleteness theorem [31]. A combination of HOL Light and Isabelle/HOL^3^ was used to check the proof by Hales of the Kepler conjecture on sphere packing. The proof was deemed too complex to be fully checked by a panel of 12 referees and this motivated Hales to establish the Flyspeck project, completed on 10 August 2014 [32], to mechanically check his proof.

It was his early unsuccessful attempts to prove programs correct using the automatic theorem prover he had implemented at Swansea that first inspired Milner to develop the ideas in the paper. These ideas have resulted in the tactic-inspired proof assistants currently being developed and used for impressive software correctness proofs such as that of the seL4 operating system [33] and the CompCert C compiler [34], which used Isabelle and Coq, respectively. The application of Milner's ideas to prove correctness extends beyond software. For example, HOL, a direct descendant of Edinburgh LCF, has been used to prove the correctness of an early ARM processor [35], Isabelle has been used to analyse the correctness of security protocols [36] and Coq to perform cryptographic proofs [37].

Proofs like those just mentioned take many person-years to perform, though much of this is time spent proving basic mathematical results. As more mathematical theories are formalized, the time needed to prove new results should decrease. Milner anticipated this cumulative development in the paper:

[…] in any realistic work with the machine as a proof assistant we expect to be working in a particular problem domain, or *theory* as we shall call it […] it should […] allow us access to those tactics that we have previously defined either of general use or pertaining to the theory in question. Furthermore, almost every interesting applied theory is founded upon more primitive theories (called its ancestor theories), and while working in any theory we expect to have access to all material pertaining to its ancestors.

An important function of the proof assistant is therefore to keep our tower of theories properly organized, allowing us […] to work in any existing theory (not only those at the top of the tower) by proving new theorems in them.

Modern proof assistants have sophisticated support for the ‘tower of theories’ and these towers are already large; as they grow, so will the efficiency of proving theorems using proof assistants.

A problem that arises both in checking pure mathematical proofs, and in performing hardware and software correctness verification proofs, is the use of different proof assistants, perhaps with different logics. For example, in the Flyspeck project, parts of the proof of the Kepler conjecture were checked using HOL and other parts checked using Isabelle. Merging separate proofs from different systems into a single coherent proof raises theoretical and practical challenges. There has been work on general frameworks for combining proofs generated using different proof assistants [38], and for combining proofs using one-off combinations of systems [39], but combining proofs from different systems remains a research area needing further study.

### (c) Growth of the MLs

The ML programing language was specifically designed to enable users of Edinburgh LCF to define functions implementing proof rules and tactics, so ML is a functional language. It is higher-order, so that tacticals for combining tactics can be defined; it has an exception mechanism to handle the inapplicability (failure) of tactics and it has a type discipline to keep unproved conjectures separate from proved theorems. It is remarkable that such a narrowly targeted language has turned out to be useful for such a wide range of applications in areas so remote from theorem proving.

ML is not emphasized in the paper, though the example tactics are written in ML; however, the language has been key to the success of tactic-based theorem proving. In §4 Milner writes

The nature of the metalanguage ML is independent of the logic that is to be used. The richness of (meta)types in ML is such that any logic can be presented within it.

The implementation of proof assistants using ML as a metalanguage for diverse logics confirms the correctness and importance of this. Milner than goes on to say

Since it is proposed to use ML with a variety of logics, and since the language has evolved somewhat since its inception, it has become important to establish a standard for it. A step towards this standard is presented in Milner 1983; though written by the present author, it represents the work of many interested researchers.

The result of this standardization, which was organized by Milner, is standard ML, the first widely used programing language with a rigorous mathematical semantics [40]. Standard ML is used to implement Isabelle and several HOL systems. It is also commonly taught as a programing language to undergraduates; several textbooks on it have been written, a popular one being by Paulson [41]. The formal semantics of standard ML has also been, and continues to be, an inspiration to programing language researchers.

Besides standard ML, the LCF metalanguage also led to OCaml [42], which is the metalanguage for Coq and one of the HOL implementations (HOL Light), and is growing in popularity in industry. The Microsoft product F# [43] was initially derived from OCaml.

## 4. Final thoughts

Milner concludes his paper with the following paragraph:

It emerges from these various studies that the method of composing proof tactics, which is illustrated in this paper on a rather simple example, not only provides a means of communicating proof methods to a machine, and of tuning them to particular needs, but also presents to mathematicians and engineers a lucid way of communicating such methods among themselves.

Developments in the past 30 years show that the idea of tactics and their implementation in a higher-order strongly typed functional programing metalanguage has had an enormous impact on ‘communicating proof methods to a machine’ for checking complex mathematical proofs [29–32] and proving the correctness of hardware and software designs [33–35]. There are many projects in progress that make it inevitable that this impact will continue to grow for the foreseeable future.

There are also signs that mathematicians and engineers are adopting Milner's ideas as ‘a lucid way of communicating such methods among themselves’. Textbooks have started to appear that use tactic-inspired proof assistants for presenting proofs [44–46]. A recent and influential research monograph [47] intriguingly states ‘much of the material presented here was actually done *first* in the fully formalized setting inside a proof assistant, and only later “unformalized” to arrive at the presentation you find before you—a remarkable inversion of the usual state of affairs in formalized mathematics’. Perhaps descendants of the ideas in the paper are just beginning to be used to explore new mathematics and to communicate the results between people.

## Footnotes

1 The notations  and *X*×*Y* denote, respectively, the set of partial functions from *X* to *Y* and the Cartesian product of *X* and *Y* —i.e. the set of pairs (*x*,*y*) with *x*∈*X* and *y*∈*Y* . The notation *X* *list* denotes the set of finite lists of members of *X*. In LCF, the empty list is denoted by [ ] and the list of *n* elements *x*_*i*_∈*X* (1≤*i*≤*n*) by [*x*_1_;⋯ ;*x*_*n*_].

2 Henceforth ‘HOL’ refers to one or all of HOL4, ProofPower or HOL Light. These proof assistants are similar, though many details differ.

3 Isabelle is a generic proof assistant. The most commonly used instances are Isabelle/HOL, which mechanizes a logic similar to (but a bit more powerful than) that of the various HOL systems and Isabelle/ZF which mechanizes first-order ZF set theory.

## Author profile

**Mike Gordon** is a member of the University of Cambridge Computer Laboratory. In 1970, he graduated BA (Mathematics) from Gonville & Caius College Cambridge; in 1973, he obtained a PhD from the University of Edinburgh, supervised by Rod Burstall, and in 1974, a Diploma in Linguistics from King's College Cambridge. In 1974, he started his first job, a research assistant to John McCarthy at the Stanford Artificial Intelligence Laboratory. In 1975, he returned to Edinburgh as a research assistant to Robin Milner on the LCF project. In 1981, he was appointed Lecturer at Cambridge and in 1996, he was promoted to Professor of Computer Assisted Reasoning, a title suggested by Milner. Gordon's research has applied and extended Milner's ideas on mechanized reasoning to both hardware and software verification. He is married to Dr Avra Cohn, Milner's second PhD student at Edinburgh.
